# The Regulation of the Small Heat Shock Protein B8 in Misfolding Protein Diseases Causing Motoneuronal and Muscle Cell Death

**DOI:** 10.3389/fnins.2019.00796

**Published:** 2019-08-02

**Authors:** Riccardo Cristofani, Paola Rusmini, Mariarita Galbiati, Maria Elena Cicardi, Veronica Ferrari, Barbara Tedesco, Elena Casarotto, Marta Chierichetti, Elio Messi, Margherita Piccolella, Serena Carra, Valeria Crippa, Angelo Poletti

**Affiliations:** ^1^Dipartimento di Scienze Farmacologiche e Biomolecolari (DiSFeB), Centro di Eccellenza Sulle Malattie Neurodegenerative, Università degli Studi di Milano, Milan, Italy; ^2^Dipartimento di Scienze Biomediche, Metaboliche e Neuroscienze, Università di Modena e Reggio Emilia, Modena, Italy; ^3^Centro Interuniversitario Sulle Malattie Neurodegenerative, Università degli Studi di Firenze, Roma Tor Vergata, Milan, Italy

**Keywords:** motoneuron diseases, amyotrophic lateral sclerosis, spinal and bulbar muscular atrophy, proteasome, autophagy, chaperones, misfolded proteins, HSPB8

## Abstract

Misfolding protein diseases are a wide class of disorders in which the aberrantly folded protein aggregates accumulate in affected cells. In the brain and in the skeletal muscle, misfolded protein accumulation induces a variety of cell dysfunctions that frequently lead to cell death. In motoneuron diseases (MNDs), misfolded proteins accumulate primarily in motoneurons, glial cells and/or skeletal muscle cells, altering motor function. The deleterious effects of misfolded proteins can be counteracted by the activity of the protein quality control (PQC) system, composed of chaperone proteins and degradative systems. Here, we focus on a PQC system component: heat shock protein family B (small) member 8 (HSPB8), a chaperone induced by harmful stressful events, including proteotoxicity. In motoneuron and muscle cells, misfolded proteins activate HSPB8 transcription and enhance HSPB8 levels, which contributes to prevent aggregate formation and their harmful effects. HSPB8 acts not only as a chaperone, but also facilitates the autophagy process, to enable the efficient clearance of the misfolded proteins. HSPB8 acts as a dimer bound to the HSP70 co-chaperone BAG3, a scaffold protein that is also capable of binding to HSP70 (associated with the E3-ligase CHIP) and dynein. When this complex is formed, it is transported by dynein to the microtubule organization center (MTOC), where aggresomes are formed. Here, misfolded proteins are engulfed into nascent autophagosomes to be degraded via the chaperone-assisted selective autophagy (CASA). When CASA is insufficient or impaired, HSP70 and CHIP associate with an alternative co-chaperone, BAG1, which routes misfolded proteins to the proteasome for degradation. The finely tuned equilibrium between proteasome and CASA activity is thought to be crucial for maintaining the functional cell homeostasis during proteotoxic stresses, which in turn is essential for cell survival. This fine equilibrium seems to be altered in MNDs, like Amyotrophic lateral sclerosis (ALS) and spinal and bulbar muscular atrophy (SBMA), contributing to the onset and the progression of disease. Here, we will review how misfolded proteins may affect the PQC system and how the proper activity of this system can be restored by boosting or regulating HSPB8 activity, with the aim to ameliorate disease progression in these two fatal MNDs.

## Introduction

Proteotoxic stress associated with aberrantly folded (misfolded) protein production is one of the factors thought to be deeply involved in the pathogenesis of several neurodegenerative diseases (NDs), including motoneuron diseases (MNDs). Amyotrophic lateral sclerosis (ALS) and spinal and bulbar muscular atrophy (SBMA) are two different types of MNDs clearly linked to the aberrant folding behavior of proteins in their conformationally unstable wild type (wt) or mutated forms (Rusmini et al., 2017). These are resistant to folding, become misfolded, and are prone to aggregate, accumulating in motoneuronal cells as well as in their surrounding (glial) or target (skeletal muscle) cells. Postmitotic cells like neurons or skeletal muscle cells are highly prone to react to misfolded protein-induced stress and mount a potent intracellular response that includes chaperone overexpression and activation of the degradative pathways. These two systems work together and are referred to the protein quality control (PQC) system. The PQC system represents the first line of defense mechanism against misfolded protein toxicity; therefore, its modulation is considered as one of the best potential targets for a possible therapeutic approach aimed to counteract MND onset and/or progression, as well as neurodegeneration.

**FIGURE 1:**
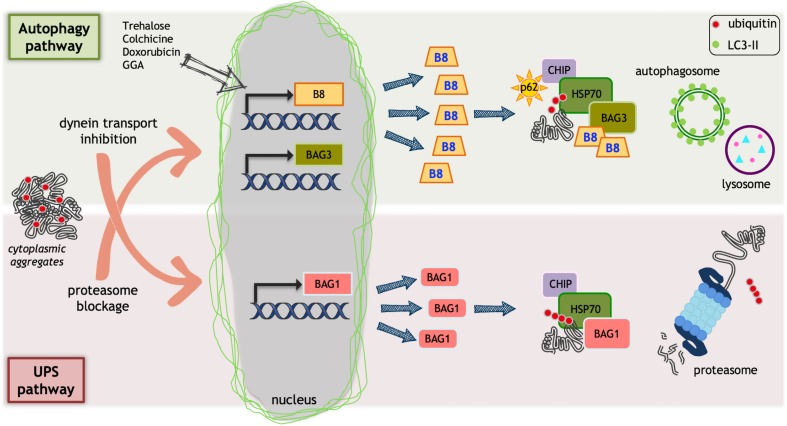
Protein quality control (PQC) system. Ubiquitin proteasome system (UPS) and autophagy could be impaired by misfolded proteins that accumulate into aggregates. Aggregates can alter dynein mediated transport or block proteasome. This causes an inefficient protein homeostasis control. As a protective mechanism, HSPB8 (B8 in the figure) and BAG3 transcription is increased by proteasome blockage that, together with their partner HSP70, facilitates misfolded proteins degradation via autophagy. Trehalose, doxorubicin, and geranylgeranylacetone (GGA) selectively increase HSPB8 transcription facilitating misfolded proteins autophagic degradation. When autophagosome formation is reduced by dynein-mediated transport inhibition, the BAG1 transcription is activated. BAG1 interacts with HSP70/CHIP and allows the degradation of misfolded proteins via UPS.

The PQC system comprises a large number of factors, which may act specifically in some subcellular compartments (i.e., chaperones located in the endoplasmic reticulum, mitochondria, lysosomes, and cytoplasm) or that are expressed in a cell and tissue specific manner. The chaperone pathways alone comprise more than 180 different chaperones and their co-regulators, while the two major degradative pathways involved in the PQC system comprise more than 600 components (in the case of the ubiquitin-proteasome system) and at least 30 different components (in the case of the autophagy system) (Hartl et al., 2011). The chaperone family comprises members that are grouped mainly on the basis of their size (small HSPs, HSP40s, HSP60s, HSP70s, HSP90s, and HSP100) and of their structure and/or function (Kampinga and Craig, 2010). Most chaperones act through the association with co-chaperones that are nucleotide exchange factors (NEFs) (Kampinga and Craig, 2010). A typical co-chaperone family is the BCL2-associated athanogene (BAG) family (Takayama and Reed, 2001). Different PQC system components, and particularly the chaperones, have been reported to be mutated and found to cause different neurodegenerative diseases. One example is represented by the small heat shock protein B8 (HSPB8), which has been found to be mutated in diseases involving motoneurons and/or muscle cells [like Charcot-Marie-Tooth type 2L disease, hereditary distal motor neuropathy type II (dHMN-II) or distal myopathy (Fontaine et al., 2006; Irobi et al., 2010; Ghaoui et al., 2016)]. This chaperone is widely expressed in almost all human tissues, and it has been proposed to be protective in ALS and SBMA (Carra et al., 2005, 2013; Crippa et al., 2010; Rusmini et al., 2013). HSPB8 is an essential member of a complex required for chaperone-assisted selective autophagy (CASA) (Figure 1). The CASA complex targets misfolded proteins to autophagy, and it is formed by two molecules of HSPB8, the HSP70 co-chaperone BAG3 (Carra et al., 2008b) and the HSP70 itself that can transiently associate to the E3-ubiquitin ligase CHIP/STUB1 (Arndt et al., 2010). Once the CASA complex is formed and associated with the misfolded target protein, the CHIP enzyme polyubiquitinates the misfolded substrate, which interacts with the autophagy receptor SQSTM1/p62. SQSTM1/p62 bridges the polyubiquitinated substrate proteins and the lipidated LC3 (LC3-II) protein, engulfing them into autophagosomes for degradation (Klionsky et al., 2016). The relevance of the CASA complex in the stress response to proteotoxicity and in neurodegenerative diseases is supported by a large body of evidence, including the finding that genetic mutations of HSPB8 (Irobi et al., 2004, 2010; Ghaoui et al., 2016) and of other three members of this complex have been linked to neurodegenerative or neuromuscular diseases. Mutant BAG3, for example, is implicated in dilated cardiomyopathy (Arimura et al., 2011), in muscular dystrophy (Selcen et al., 2009) and in giant axonal neuropathy (Jaffer et al., 2012), and STUB1/CHIP1 has been found mutated in Gordon Holmes syndrome (multisystemic neurodegeneration (Hayer et al., 2017) and in spinocerebellar ataxia 48 (SCA48) (Genis et al., 2018), while the protein has been reported to be destabilized in SCA16 in which six different variants have been reported (Pakdaman et al., 2017; Kanack et al., 2018). In addition, a missense mutation in the ubiquitin ligase domain of CHIP has been involved in the pathogenesis of spinocerebellar autosomal recessive 16 (SCAR16) (Shi et al., 2013, 2018). Finally, SQSTM1/p62 has been found to be mutated in some familial forms of ALS (fALS) (Fecto et al., 2011; Teyssou et al., 2013).

## The Role of HSPB8 in the Selection of the Proper Degradative System for Misfolded Proteins in Motoneuron Diseases

As mentioned in the introduction, ALS and SBMA are typically considered to be protein misfolding diseases, in which unstable proteins or their mutated forms tend to aggregate impairing motoneuronal or muscle functions.

From a clinical point of view, the two diseases present some difference, especially in the type of motoneurons affected. ALS is characterized by the loss of both upper and lower motoneurons and the regions affected are the brain motor cortex, the brainstem and anterior horns spinal cord motoneurons. In some fALS, alteration in the fronto-temporal regions are present and correlates with a mixed phenotype involving motor dysfunction and frontolateral temporal dementia (FLTD) (Robberecht and Philips, 2013). In ALS, not only motoneurons are affected but also glial cells [astrocytes (Trotti et al., 1999; Boillée et al., 2006; Nagai et al., 2007), oligodendrocytes (Philips et al., 2013), Schwann cells (Lobsiger et al., 2009; Turner et al., 2010) and cells of the inflammatory response, like microglia (Philips and Robberecht, 2011)]. Recent data suggest that skeletal muscle cells can also be directly involved in disease onset and progression (Musarò, 2010; Onesto et al., 2011; Galbiati et al., 2014). Conversely, in SBMA, which is characterized by a much slower progression rate compared to ALS, the motoneurons affected are only the lower motoneurons in the bulbar region and in the anterior horns of the spinal cord. The motor and frontal cortex remain unaffected, and there are no clinical signs of dementia in SBMA patients. No signs of neuroinflammation involving microglial cells or of alteration in glial cells have been reported in SBMA, indicating that microglia is not involved (La Spada et al., 1991; Fischbeck, 1997; Soraru et al., 2008; Boyer et al., 2013; Malena et al., 2013; Cortes et al., 2014a; Lieberman et al., 2014). Interestingly, SBMA patients display sensory alteration due to the loss of dorsal root ganglia (DRG) sensory neurons. Finally, there are clear data indicating that, in SBMA, the skeletal muscle cells and several reproductive tissues containing androgen-target cells are directly affected (La Spada et al., 1991; Fischbeck, 1997; Adachi et al., 2005; Soraru et al., 2008; Boyer et al., 2013; Malena et al., 2013; Chua et al., 2014; Cortes et al., 2014a; Lieberman et al., 2014; Halievski et al., 2015; Sahashi et al., 2015; Xu et al., 2016, 2018).

ALS mainly appears as sporadic forms (sALS), but about 10% of the cases are inherited (fALS) and several mutated genes have already been described (Table 1; Cook and Petrucelli, 2019). Of note, mutations in these genes often result in the production of pathological misfolded/aggregating-prone proteins (Oskarsson et al., 2018). Moreover, these genes code for proteins that, even in their wt form, tend to be conformationally unstable (Table 1) forming misfolded species, which for unknown reasons aberrantly accumulate in sALS, causing cell death. This observation suggests the existence of common pathogenic mechanisms in fALS and sALS (Neumann et al., 2006; Daoud et al., 2009; Ju et al., 2009; Bosco and Landers, 2010; Tresse et al., 2010; Robberecht and Philips, 2013; Taylor et al., 2016). SBMA appears only in an inherited form, and it is associated to a CAG triplet repeat sequence expansion in exon 1 of the gene coding for the androgen receptor (AR). The coded AR protein contains a translated polyglutamine (polyQ) tract which confers toxicity to the ARpolyQ. Notably, this toxicity appears only when the ARpolyQ is bound to its endogenous ligands testosterone or dihydrotestosterone (DHT) (La Spada et al., 1991; Stenoien et al., 1999; Simeoni et al., 2000; Katsuno et al., 2002, 2003; Poletti, 2004).

**TABLE 1:** List of genes related to ALS and SBMA.

**Gene**	**Name**	**Protein**	**Protein function**	**Aggregating/misfolded species**
**ALS**				
ALS2	Alsin	ALS2	Vesicle trafficking	
ANG	Angiogenin	ANG	Ribonuclease	
ANXA11	Annexin A11	ANXA11	Vesicle trafficking, apoptosis, exocytosis, and cytokinesis	Mutated
ATXN2	Ataxin 2	ATXN2	Endocytosis/RNA metabolism	Mutated; wt
C21orf2	Chromosome 21 Open Reading Frame 2	C21orf2	Mitochondrial dysfunction, cytoskeletal dynamics	
C9orf72	Chromosome 9 Open Reading Frame 72	C9orf72	Possible guanine nucleotide exchange factor-involved in autophagy	Dipeptide-repeat (DPR) proteins generated by ATG- independent transcription of the ALS/FTD-related abnormal GGGGCC expansion
CCNF	Cyclin F	CCNF	Catalyzes ubiquitin transfer to substrates for UPS degradation	
CHCHD10	Coiled-coil-helix-coiled-coil-helix domain-containing protein 10	CHCHD10	Mitochondrial protein	
CHMP2B	Charged multivesicular body protein 2B	CHMP2B	Protein degradation	
DLTNl	Dynactin subunit 1	DCTN1	Component of dynein motor complex	
EWSR1	Ewing Sarcoma breakpoint region 1	EWSR1	RNA/DNA binding protein	Mutated; wt is intrinsically prone to aggregation
FIG4	Phosphoinositide 5-phosphatase	FIG4	Protein degradation	
FUS	Fused in Sarcoma	FUS	RNA binding protein	Mutated; wt FUS sequestered into pathological aggregates
HNRNPA1	Heterogeneous nuclear ribonucleoprotein Al	HNRNPA1	RNA-binding protein	Mutated
HNRNPA2/B1	Heterogeneous nuclear ribonucleoprotein A2/B1	HNRNPA2/B1	RNA-binding protein	Mutated
KIF5A	Kinesin family member 5A		Microtubule-based motor protein	
MATR3	Matrin 3	MATR3	RNA-binding protein	Mutated and wt (rare inclusions)
NEFH	High molecular weight neurofilaments	NFH	Cytoskeletal component	Mutated
NEK1	NIMA Related Kinase 1	NEK1	Cytoskeletal dynamics	
OPTN	Optineurin	OPTN	Autophagy adaptor	wt OPTN sequestered into pathological aggregates
PFN1	Profilin 1	PFN1	Actin binding protein	Mutated
SETX	Senataxin	SETX	RNA/DNA helicase	
SOD1	Cu-Zn superoxide dismutase 1	SOD1	Superoxide dismutase	Mutated; oxidized wild type (wt) SOD1
SPG11	Spatacsin	SPG11	DNA damage repair	
SQSTM1	Sequestosome 1	SQSTM1	Autophagy adaptor	Mutated; wt SQSTM1 sequestered into pathological aggregates
TAF15	TATA box binding protein associated factor 15	TAF15	RNA-binding protein	Mutated; wt is intrinsically prone to aggregation
TARDBP	TAR DNA Binding Protein	TDP-43	RNA-binding protein	Mutated TDP-43s; phopshorylated wt TDP-43; wt full-length TDP-43 and its C-terminal fragments (TDP-35andTDP-25)
TBK1	Serine/threonine-protein kinase TBK1	TBK1	Innate immune response, autophagy, inflammation and cell proliferation	
TIA1	T cell-restricted intracellular antigen 1	TIA1	RNA-binding protein	Mutated TIA1 showed altered stress granules dynamics
TUBA4A	Tubulin alpha 4a	TUBA4A	Microtubule subunit	Mutated
UBQLN2	Ubiquilin 2	UBQLN2	Autophagy adaptor	Mutated; wt UBQLN2 sequestered into pathological aggregates
VAPB	VAMP/synaptobrevin-associated protein	VAPB	ER-membrane protein	Mutated
VCP	Valosin containing protein	VCP	Ubiquitin segregase	
**SBMA**				
AR	Androgen receptor	AR	Nuclear receptor that mediates male hormones effects	Mutant polyQ, ligand-dependent

*The table illustrates the genes found mutated in ALS (upper part) and SBMA (lower part), listed in alphabetical order. For each gene, the name, the coded protein and its cellular function are indicated, if known. In the last column, the aggregation/misfolding propensity of the protein is depicted, specifying the affected species [wild-type (wt) and/or mutated]. In green the ALS genes that are most frequently mutated and collectively account for 60–70% of fALS and 10% of sALS cases.*

At present, it remains unclear if protein misfolding is the primary toxic event in ALS, or if it reflects alteration of specific intracellular pathways (e.g., alterations of the PQC system). Several recent data underline that autophagy dysfunction is implicated in ALS (Valenzuela et al., 2018; Evans and Holzbaur, 2019; Nguyen et al., 2019) and SBMA (Cortes et al., 2014b), but its role in diseases is still debated (Chua et al., 2014; Cristofani et al., 2017, 2018). On one side, autophagy defects are clearly involved, since different genes found mutated in ALS code for proteins of the autophagic system, like SQSTM1/p62, OPTN, VCP, UBQLN2, TBK1 and C9orf72 (Table 1; Nguyen et al., 2019). Altered autophagic flux has furthermore been observed in ALS patients and confirmed in both cell and animal ALS models. On the other hand, an excessive autophagy seems to be related to the disease (Nguyen et al., 2019). Pharmacological manipulations of autophagy performed in different ALS models confirm this dual role of this degradative pathway in ALS (Valenzuela et al., 2018). For example, the treatment of SOD1-G93A mice with the mTOR-independent autophagy stimulator trehalose significantly prolonged life span and attenuated the disease signs, decreased SOD1 aggregates and enhanced motoneuron survival (Castillo et al., 2013; Zhang et al., 2014; Li et al., 2015). On the contrary, the mTOR-independent autophagy stimulator rilmenidine worsened motor neurons degeneration and symptom progression in SOD1-G93A mice (Perera et al., 2018). Similar results were observed also after treatment with the mTOR-dependent autophagy stimulator rapamycin, which exacerbated the pathological process of SOD1-G93A mice by accelerating the motor neurons degeneration, shortening the life span causing mitochondrial impairment and caspase-3 activation (Zhang et al., 2011).

With regards to SBMA, accumulation of autophagosome and reduced autophagic flux have been observed in cell and animal models of SBMA (Rusmini et al., 2010, 2013; Cortes et al., 2014a,b; Giorgetti et al., 2015; Cristofani et al., 2017). Interestingly, while the wild type AR positively regulates the activity of the transcription factor EB (TFEB, a master regulator of autophagy and lysosomal biogenesis), the mutant ARpolyQ interferes with TFEB activity, reducing its control on target genes and thus leading to autophagy dysregulation (Cortes et al., 2014b). Otherwise, in SBMA mice models, autophagy is upregulated in skeletal muscle during disease progression, indicating that tissue-specific aberrant activation of TFEB signaling might contribute to SBMA pathogenesis (Cortes et al., 2014a,b; Rusmini et al., 2015; Rocchi et al., 2016). In addition, a very recent observation suggests that alternative autophagic pathways can also be dysregulated. Indeed, the charged multivesicular body protein 7 (Chmp7) gene, which codes for an ESCRT-III related protein involved in autophagic flux and the endo-lysosomal sorting pathway is downregulated in induced pluripotent stem cells (iPSCs) derived from SBMA patients as well as in SBMA mice models even before disease onset. This suggests that CHMP7 may play a primary role protein in autophagic flux alteration observed in SBMA (Malik et al., 2019).

Several studies have proposed that HSPB8 is implicated both in ALS and SBMA, possibly by acting as a protective agent against disease onset and/or progression (Carra et al., 2005, 2008b, 2010; Crippa et al., 2010, 2013a,b, 2016a,b; Rusmini et al., 2013, 2019; Cristofani et al., 2017, 2018; Cicardi et al., 2018). It has been shown that, during disease manifestations and at the end stage of disease, HSPB8 is highly expressed in the spinal cord of the SOD1-G93A ALS mouse model and in spinal cord specimens of ALS patients (Anagnostou et al., 2010; Crippa et al., 2010). Moreover, HSPB8 was found upregulated in the lateral tract astrocytes of patients with short disease duration (Gorter et al., 2018). In the SOD1-G93A ALS mouse model, the high levels of HSPB8 are confined specifically in anterior horn spinal cord motoneurons that survive at the end stage of disease (Crippa et al., 2010). These data are of great interest, since in non-transgenic (NTg) normal mice, the expression of HSPB8 within the spinal cord typically decreases with age (Crippa et al., 2010). Thus, these motoneurons are potentially more vulnerable to the toxicity of mutant misfolded proteins, and HSPB8 overexpression in motoneurons of the SOD1-G93A affected mice could represent a cell response to damages induced by misfolded proteins. The increased HSPB8 levels may enhance proteotoxic stress tolerance of these surviving motoneurons.

In NTg mice, HSPB8 is also present at high levels in muscles; instead, in the SOD1-G93A ALS or SBMA AR113Q mouse models, HSPB8 expression is robustly increased paralleling disease progression (Crippa et al., 2013a,b; Rusmini et al., 2015). This is not unexpected, since skeletal muscle cells are a direct target of misfolded protein toxicity in both ALS and SBMA; therefore, the enhanced production of HSPB8 may serve to protect this tissue during disease progression. This interpretation is suggested by the finding that, in TDP-43 ALS *Drosophila melanogaster* models (TDP-43, TDP-35, and TDP-25), the overexpression of the HSPB8 functional ortholog (*HSP67Bc)* protects from misfolded protein toxicity, while its downregulation has the opposite effect (Crippa et al., 2016a). In line with these data is the observation that a viral homologue of HSPB8—the protein ICP10PK, carried by the herpes simplex virus type 2 (HSV-2)—when used to infect the SOD1-G93A ALS rat model is able to delay symptom onset and reduce the progression of the disease, thus enhancing the overall survival of the mice (Aurelian, 2012; Aurelian et al., 2012). Whether ICP10PK exerts similar functions in PQC compared to HSPB8 or whether its protective activity is due to other mechanisms that are not related to PQC and autophagy is still unknown.

Collectively, the data obtained using ALS and SBMA animal models corroborate the notion that HSPB8 is protective against misfolded protein toxicity in these diseases.

The observations performed at cellular and molecular levels parallel the animal data, showing that HSPB8 has a potent antiaggregant activity, and facilitates the removal of aggregating misfolded proteins from a variety of neuronal and muscle models of ALS and SBMA. For example, HSPB8 reduces the accumulation of several polyQ proteins, like polyQ of the mutant huntingtin and ataxin-3, as well as of the beta-amyloid protein, of the alpha-synuclein, and of a large number of ALS-associated mutant proteins (like the mutant SOD1-G93A or the mislocalized TDP-43 C-terminal fragments (TDP-35 and TDP-25) and the abnormally translated dipeptides (DPRs) produced from the expanded GGGGCC repeated sequence of the *C9orf72* gene causing ALS and/or FLTD) (Chavez Zobel et al., 2003; Wilhelmus et al., 2006; Carra et al., 2008a,b; Crippa et al., 2010, 2016b; Bruinsma et al., 2011; Seidel et al., 2012; Rusmini et al., 2013; Cristofani et al., 2017, 2018; Cicardi et al., 2018).

Very recently, it has been shown that HSPB8 is also able to maintain a correct dynamic behavior of stress granules (SGs), membraneless ribonucleoprotein (RNP) complexes that form via liquid-liquid phase separation (LLPS) (Banani et al., 2017).

In the past decade, researchers have sought to understand the principles that regulate the formation and dissolution of SGs, due to their potential implication in a number of neurological disorders [e.g., ALS, FLTD, Alzheimer’s disease (AD), etc.] (Elden et al., 2010; Taylor et al., 2016). Similar to other types of membraneless organelles, such as PML bodies (Banani et al., 2017), SGs are highly dynamic and are induced by stress conditions, including oxidative stress, viral infection, and temperature upshift, but they tend to dissolve upon stress relief (Anderson and Kedersha, 2002). While SGs tend to dissolve rapidly after stress relief in healthy cells, SGs were reported to persist for longer time in cell models of ALS that express mutated, disease-causing forms of TDP-43, FUS, TIA-1, and hnRPNA1. TDP-43, FUS, TIA-1, and hnRPNA1 are all RNA-binding proteins recruited inside SGs. These RBPs can form liquid droplets *in vitro* that are unstable and can mature with time into amyloid-like aggregates (Molliex et al., 2015; Patel et al., 2015; Mackenzie et al., 2017). The ALS-associated mutated forms of TDP-43, FUS, TIA-1, and hnRPNA1 accelerate the conversion of the liquid droplets into a solid aggregated-like state *in vitro*, while they all confer rigidity to SGs in ALS cell models, thereby delaying their disassembly kinetics and favoring the co-aggregation of SGs with other misfolded aggregate-prone proteins (Ganassi et al., 2016; Taylor et al., 2016; Mateju et al., 2017). These observations have therefore, suggested the hypothesis that SGs play an important role in ALS. Recent data obtained using light-inducible SGs (or OptoGranules) showed that the repetitive induction of SGs leads to their conversion into neuronal aggregates that become enriched for phosphorylated TDP-43 forms, ubiquitin and SQSTM1/p62 (Zhang et al., 2019), typical hallmark of inclusions found in sALS and fALS (Neumann et al., 2009). Thus, SGs may contribute to the formation of the neuronal pathological inclusions (Zhang et al., 2019).

So far, two mechanisms that decrease SG dynamics have been identified: (1) the presence of unstable aggregate-prone RBPs (Molliex et al., 2015; Patel et al., 2015) and (2) the accumulation inside SGs of misfolded proteins [including defective ribosomal products (DRiPs) and ALS/FLTD-linked DPRs] (Ganassi et al., 2016; Lee et al., 2016; Mateju et al., 2017). Enhanced clearance of misfolded proteins, DRiPs or DPRs may, therefore, indirectly facilitate disassembly of SGs, restoring their physiological dynamics. Enhanced clearance of aberrant SGs may furthermore exert protective functions. Indeed, the accumulation of misfolded proteins inside SGs is prevented by the action of molecular chaperones such as VCP and the HSPB8-BAG3-HSP70 complex, which target misfolded proteins, DRiPs and DPRs to degradation (Verma et al., 2013; Seguin et al., 2014; Ganassi et al., 2016). In addition, ZFAND, VCP, and SQSTM1/p62 facilitate the degradation of persisting aberrant SGs via autophagy and the proteasome, respectively.

The formation of cytoplasmic aggregates containing phosphorylated TDP-43 may arise also with SG-independent mechanisms, and can be induced by fibrillar fragments of aggregated TDP-43 itself or FUS (Gasset-Rosa et al., 2019). These aggregates sequester nuclear transport factors, impairing the nucleocytoplasmic shuttling (Freibaum et al., 2015; Jovicic et al., 2015; Boeynaems et al., 2016; Woerner et al., 2016; Kim and Taylor, 2017; Chou et al., 2018). Considered together, these studies demonstrate that SG-dependent and SG-independent mechanisms contribute to ALS disease progression. They likewise suggest that SG-dependent and SG-independent mechanisms may converge so as to favor the formation of the pathological inclusions. Consequently, approaches that limit the cytoplasmic accumulation of aggregated TDP-43, such as overexpression of HSPB8 or induction of autophagy, may exert beneficial effects by acting both on SG-dependent and independent TDP-43 aggregates.

From the molecular point of view, the mechanism by which HSPB8 blocks the accumulation of misfolded proteins in cells affected in these MNDs is intriguing. As mentioned above, HSPB8 is a crucial component of the CASA complex. The intracellular levels of HSPB8 appear not to be sufficient to handle the large amounts of misfolded proteins that accumulate under certain stress conditions. In fact, the single overexpression of HSPB8 is able to restore a sufficient clearance of misfolded proteins, preventing their aggregation in cells. It is thus not surprising that the main activity of HSPB8 is to act as an autophagy facilitator. The first proof for this action comes from the observation that HSPB8 strongly interacts with the Ile-Pro-Val (IPV) domains of BAG3 forming a stable complex (Fuchs et al., 2010, 2015). Moreover, BAG3 might use the IPV domains to interact also with other HSPBs, like HSPB1, HSPB2 (Morelli et al., 2017), HSPB5 (αB-crystallin) (Hishiya et al., 2011), and HSPB6 (Fuchs et al., 2009; Rauch et al., 2017).

Like HSPB8, other members of the mammalian HSPB family have been linked to ALS progression. Indeed, two HSPB1 variants have been reported in a cohort of unrelated Italian ALS patients, while the loss of chaperone-like activity was demonstrated in one of the mutant proteins (Capponi et al., 2016). Like HSPB8, also HSPB1, together with αB-crystallin, were found upregulated in the spinal cords of different symptomatic mutant SOD1 mice (G37R, G93A, G85R, H46R/H48Q), compared to control (Vleminckx et al., 2002; Wang et al., 2003). Notably, we observed in two similar SOD1-G93A mice strains that were characterized by a different progression rate (fast vs slow progression) that low basal expression of the αB-crystallin correlates with a fast progressing phenotype, whereas high αB-crystallin levels correlate with a more slowly progressing phenotype (Marino et al., 2015). This observation is indicative of a protective role of αB-crystallin in these animals. *In vitro* experiments also support this hypothesis, since both αB-crystallin and HSPB1 overexpression are able to suppress SOD1 aggregation (Yerbury et al., 2013). Focusing on HSPB1, its role in ALS is rather controversial; for example, HSPB1 overexpression is beneficial when tested in different SOD1-based ALS cell models (Patel et al., 2005; Krishnan et al., 2006; An et al., 2009; Yerbury et al., 2013; Heilman et al., 2017), but animal model experiments did not confirm this protective role. In its work, Krishnan et al. (2008) showed that the ubiquitous over-expression of human HSPB1 in SOD1-G93A mice [double transgenic SOD1(G93A)/hHSPB1 mice] did not affect disease duration, progression, motor neuron degeneration or SOD1 aggregation, although hHSPB1 overexpression alone (single transgenic hHSPB1 mice) protected against spinal cord ischemia (Krishnan et al., 2008). Slightly different results were obtained by Sharp et al. (2008), where the SOD1(G93A)/HSPB1 double transgenic mice showed an improvement in some pathological parameters compared to SOD1-G93A mice; this protective activity was present at the early stage of disease but was lost at later stages (Sharp et al., 2008). Interestingly, in these mice the expression of hHSPB1 protein in affected cells decreased during disease progression, although mRNA levels remained unchanged, and so far no explanation for this phenomenon have been provided, but this reduced translation/enhanced clearance of HSPB1 may help to explain the lack of protection at late stage of disease.

Part of the protective activity of HSPBs against neuronal loss may be due to their ability to interact with BAG3. In addition to the binding with different HSPBs, BAG3, with its BAG domain, can bind directly the HSP70 already involved in a heterodimer with CHIP (Figure 1). When HSPB8 binds, the CASA complex is formed, and this allows misfolded protein recognition. BAG3 is a scaffold protein, which also contains a PXXP motif for the binding to dynein, and this interaction is reinforced by the 14-3-3 protein, which binds in close proximity to the PXXP motif (McCollum et al., 2009; Merabova et al., 2015). Dynein has now the capability to move retrogradely the CASA complex with the misfolded protein (polyubiquitinated by CHIP) along microtubules to the MTOC, where aggresomes are formed and autophagosomes assembled.

Dynein mediated transport has been involved in ALS pathogenesis: (i) dynein has been detected in SOD1 aggregates and (ii) alteration of retrograde transport is present in transgenic SOD1-G93A mice even if the legs at odd angles (Loa) mutation in cytopasmic dynein could be protective in transgenic SOD1-G93A mice where it delays disease onset and extends the life span (Kieran et al., 2005; Zhang et al., 2007; Bilsland et al., 2010; El-Kadi et al., 2010). Moreover, DCTN1 and KIF5A motor protein and TUBA4, NEFH, and NEK1 cytoskeleton proteins are related to ALS (Table 1).

The polyubiquitinated misfolded proteins are then/finally recognized by the autophagy receptor (like SQSTM1/p62) and engulfed by the lipidated LC3-II into nascent autophagosome for clearance [see (Rusmini et al., 2017)]. This mechanism has been initially elucidated in physiological condition in muscle fiber subjected to extensive physical exercise, in which large amounts of damaged (carbonylated, nitrosylated, etc.) proteins are generated. Indeed, the CASA complex is essential for Z-disk maintenance in skeletal muscle (Arndt et al., 2010; Ulbricht et al., 2015). We proved that this mechanism takes place also in motoneurons, in pathological conditions due to the presence of ALS or SBMA-associated misfolded proteins (Crippa et al., 2010, 2016a,b; Rusmini et al., 2013; Cristofani et al., 2017, 2018; Cicardi et al., 2018). The CASA complex may also involve HSP40 (particularly DNAJB6) (Sarparanta et al., 2012), which acts as an HSP70 co-chaperone to block misfolded protein aggregation (Hageman et al., 2010). Like several members of the CASA complex, mutation in *DNAJB6* has been identified in human diseases linked to aberrant protein aggregation, like Limb-girdle muscular dystrophies (LGMDs) (Sandell et al., 2016). Of note, DNAJB6 aggregates in muscles of LGMD patients and sequesters BAG3, HSPB8, HSP70 and CHIP in inclusions of different sizes present in the cell cytoplasm. Curiously, in the same specimens DNAJB6 was also present in nuclear aggregates that were positive exclusively for HSPB8 (Sato et al., 2013), leading to the hypothesis that these two proteins may interact during CASA complex formation earlier, or at a different cell location, than with the other members of the CASA complex.

Even more intriguing is the existence of an alternative way to escape HSPB8/BAG3 recognition when the function of the CASA complex is blocked. This way reroutes substrates, including misfolded proteins, from autophagic to proteasomal degradation. For example, if the dynein mediated transport of the CASA complex is blocked, the heterodimer HSP70-CHIP does not interact with HSPB8-BAG3, but preferentially associates to an alternative interactor: BAG1 (Cristofani et al., 2017). Like BAG3, BAG1 is a NEF/BAG co-chaperone of HSP70, which after interacting with HSP70-CHIP routes cargoes, including misfolded proteins, to the proteasome (Arndt et al., 2010; Ulbricht et al., 2013; Figure 1). It is relevant to note that the blockage of the CASA complex transport to MTOC induces the transcriptional activation of the BAG1 gene. On the other hand, the blockage of proteasome induces the *de novo* synthesis of HSPB8 and BAG3 (Yew et al., 2005; Carra, 2009; Crippa et al., 2010; Carra et al., 2013; Cristofani et al., 2017). Therefore, this connected transcriptional regulation gives rise to a fine-tuned equilibrium between autophagy and proteasome and allows the selection of the proper degradative pathway during different types of proteotoxic stresses, which may differentially impact on one of the two systems (Arndt et al., 2010; Crippa et al., 2010; Behl, 2011, 2016; Gamerdinger et al., 2011a,b; Lilienbaum, 2013; Xu et al., 2013; Jia et al., 2014; Minoia et al., 2014; Merabova et al., 2015; Cristofani et al., 2017). An unbalanced equilibrium between these two systems may thus account for the aberrant accumulation of misfolded proteins in MNDs and in NDs in general (Kakkar et al., 2014; Ciechanover and Kwon, 2015; Nikoletopoulou et al., 2015; Senft and Ronai, 2015; Xilouri and Stefanis, 2015).

The involvement of other HSPs, in particular HSP70 and HSP90, in ALS has been deeply reviewed in Kalmar and Greensmith (2017) and Lackie et al. (2017).

## Identification of Small Molecules that Regulate HSPB8 Expression in Mnd-Affected Cells

In line with the data summarized above, it would be of interest to determine whether the expression of HSPB8, which is sufficient to restore autophagy, can be enhanced by acting at the level of its gene transcription, thus preventing its role of limiting factor for the CASA complex. Several small molecules have already been identified to act as HSPB8 inducers and could be an opportunity to be tested in clinical trials in MNDs. In a large, high-throughput screening, based on a luciferase reporter controlled by the human HSPB8 promoter, we were able to find several FDA-approved drugs capable of modulating HSPB8 gene expression. Among the list of hits, we selected colchicine and doxorubicin and showed that these compounds enhance the clearance of insoluble TDP-43 species (hallmark for ALS) in a HSPB8-dependent (even if not exclusive) manner (Crippa et al., 2016b). Since colchicine is a safe drug with a well-established pharmacokinetic, it is now under investigation in a phase II clinical trial on a large cohort of ALS patients (Mandrioli et al., 2019).

Other small molecules that are able to induce HSPB8 expression have been characterized. One is trehalose, a non-toxic natural compound well-known for its ability to induce autophagy (Rusmini et al., 2013), whose mechanism of action has been recently unraveled. TFEB is the main mediator of the effects of trehalose. Trehalose treatment correlates with the activation of the calcineurin/TFEB pathway by a rapid and transient lysosomal membrane permeabilization and, possibly, by lysosomal calcium release. This event triggers the induction of TFEB target genes leading to specific removal of damaged lysosomes by autophagy (called lysophagy) and the restoration of normal lysosomal homeostasis (Rusmini et al., 2019). Trehalose has been proven, both in cell and animal models, to be very efficient in the removal of misfolded proteins in many different NDs (Tanaka et al., 2004; Davies et al., 2006; Rodriguez-Navarro et al., 2010; Perucho et al., 2012; Schaeffer and Goedert, 2012; Castillo et al., 2013; Du et al., 2013; Sarkar et al., 2014; Zhang et al., 2014; He et al., 2016).

Estrogens and selective estrogen receptor modulators (SERMs) are also potent activators of HSPB8 expression (Sun et al., 2007; Piccolella et al., 2017), and this might help to explain the existence of gender differences in the risk to develop some age-related forms of NDs (Villa et al., 2016).

Geranylgeranylacetone (GGA), also known as teprenone, which is an inducer of several HSPs, is also a potent upregulator of HSPB8 expression, and has been proven to be able to decrease the formation of amyloid oligomer and aggregates in desmin-related cardiomyopathy (Sanbe et al., 2009).

Finally, one of the best known regulators of HSPB8 (and BAG3) is the NF-κB transcription factor, which is generally activated in the recovery period that follows a heat shock (Nivon et al., 2012). Even if the control of this pathway is still obscure, it is expected that modulators of the NF-kB pathway may also influence the expression of HSPB8 in cells. Whether or not this approach may have therapeutic perspectives remains to be elucidated.

## Conclusion

In conclusion, biochemical and immunohistochemical data obtained by using cell and animal (mouse and Drosophila) models of ALS and other neurodegenerative diseases support the interpretation that HSPB8 has a prominent role in counteracting the toxicity of misfolded proteins in MNDs and may be fundamental in the maintenance of the delicate equilibrium that regulates the routing of proteins to autophagy and to the proteasome. This is further suggested by the finding that HSPB8 and BAG3 are upregulated in the postmortem tissues from patients affected by several types of protein conformational diseases, specifically in the regions interested by neurodegeneration (Anagnostou et al., 2010; Seidel et al., 2012). HSPB8 may act as a limiting factor in this context, and its transcriptional induction or functional activation with small molecules may serve as a potential approach to counteract the onset and/or progression of these devastating NDs. Despite the fact that how HSPB8 works at the molecular level and how its expression is regulated have not yet fully been elucidated, the current literature highlights its relevance in several NDs, prompting us to investigate how to exploit the functions of this chaperone against neurodegeneration. The availability of safe drugs that are able to induce HSPB8 expression in MNDs may be the first step to clarify its potential protective role in these diseases.

## Author Contributions

PR, RC, MG, VC, and AP designed and wrote the manuscript, and critically discussed all the sections of this minireview. RC prepared the figure. MEC, VF, BT, EC, MC, EM, MP, and SC critically revised the manuscript and figure. All authors have provided final approval of the version to be published.

## Conflict of Interest Statement

The authors declare that the research was conducted in the absence of any commercial or financial relationships that could be construed as a potential conflict of interest.

## References

[B1] Adachi H. Katsuno M. Minamiyama M. Waza M. Sang C. Nakagomi Y. (2005). Widespread nuclear and cytoplasmic accumulation of mutant androgen receptor in SBMA patients. *Brain* 128(Pt 3), 659–670. 10.1093/brain/awh381 15659427

[B2] An J. J. Lee Y. P. Kim D. W. Sohn E. J. Jeong H. J. Kang H. W. (2009). Transduced HSP27 protein protects neuronal cell death by enhancing FALS-associated SOD1 mutant activity. *BMB Rep.* 42 136–141. 10.5483/bmbrep.2009.42.3.136 19335999

[B3] Anagnostou G. Akbar M. T. Paul P. Angelinetta C. Steiner T. J. de Belleroche J. (2010). Vesicle associated membrane protein B (VAPB) is decreased in ALS spinal cord. *Neurobiol. Aging* 31 969–985. 10.1016/j.neurobiolaging.2008.07.005 18701194

[B4] Anderson P. Kedersha N. (2002). Visibly stressed: the role of eIF2, TIA-1, and stress granules in protein translation. *Cell Stress Chaperones* 7 213–221. 10.1379/1466-1268(2002)007<0213:vstroe<2.0.co;2 12380690 PMC514820

[B5] Arimura T. Ishikawa T. Nunoda S. Kawai S. Kimura A. (2011). Dilated cardiomyopathy-associated BAG3 mutations impair Z-disc assembly and enhance sensitivity to apoptosis in cardiomyocytes. *Hum. Mutat.* 32 1481–1491. 10.1002/humu.21603 21898660

[B6] Arndt V. Dick N. Tawo R. Dreiseidler M. Wenzel D. Hesse M. (2010). Chaperone-assisted selective autophagy is essential for muscle maintenance. *Curr. Biol.* 20 143–148. 10.1016/j.cub.2009.11.022 20060297

[B7] Aurelian L. (2012). “The HSV-2 gene ICP10PK: a future in the therapy of neurodegeneration,” in *From the Hallowed Halls of Herpesvirology: A Tribute to Bernard Roizman*, eds Blaho J. A. Baines J. D. (Singapore: World Scientific), 1–21. 10.1142/9789814338998_0001

[B8] Aurelian L. Laing J. M. Lee K. S. (2012). H11/HspB8 and its herpes simplex virus type 2 homologue ICP10PK share functions that regulate cell life/death decisions and human disease. *Autoimmune Dis.* 2012:395329. 10.1155/2012/395329 23056924 PMC3463903

[B9] Banani S. F. Lee H. O. Hyman A. A. Rosen M. K. (2017). Biomolecular condensates: organizers of cellular biochemistry. *Nat. Rev. Mol. Cell Biol.* 18 285–298. 10.1038/nrm.2017.7 28225081 PMC7434221

[B10] Behl C. (2011). BAG3 and friends: co-chaperones in selective autophagy during aging and disease. *Autophagy* 7 795–798. 10.4161/auto.7.7.15844 21681022

[B11] Behl C. (2016). Breaking BAG: the co-chaperone BAG3 in health and disease. *Trends Pharmacol. Sci.* 37 672–688. 10.1016/j.tips.2016.04.007 27162137

[B12] Bilsland L. G. Sahai E. Kelly G. Golding M. Greensmith L. Schiavo G. (2010). Deficits in axonal transport precede ALS symptoms in vivo. *Proc. Natl. Acad. Sci. U.S.A.* 107 20523–20528. 10.1073/pnas.1006869107 21059924 PMC2996651

[B13] Boeynaems S. Bogaert E. Michiels E. Gijselinck I. Sieben A. Jovicic A. (2016). Drosophila screen connects nuclear transport genes to DPR pathology in c9ALS/FTD. *Sci. Rep.* 6:20877. 10.1038/srep20877 26869068 PMC4751451

[B14] Boillée S. Vande Velde C. Cleveland D. W. (2006). ALS: a disease of motor neurons and their nonneuronal neighbors. *Neuron* 52 39–59. 10.1016/j.neuron.2006.09.018 17015226

[B15] Bosco D. A. Landers J. E. (2010). Genetic determinants of amyotrophic lateral sclerosis as therapeutic targets. *CNS Neurol. Disord. Drug Targets* 9 779–790. 10.2174/187152710793237494 20942785

[B16] Boyer J. G. Murray L. M. Scott K. De Repentigny Y. Renaud J. M. Kothary R. (2013). Early onset muscle weakness and disruption of muscle proteins in mouse models of spinal muscular atrophy. *Skelet. Muscle* 3:24. 10.1186/2044-5040-3-24 24119341 PMC3852932

[B17] Bruinsma I. B. Bruggink K. A. Kinast K. Versleijen A. A. Segers-Nolten I. M. Subramaniam V. (2011). Inhibition of alpha-synuclein aggregation by small heat shock proteins. *Proteins* 79 2956–2967. 10.1002/prot.23152 21905118

[B18] Capponi S. Geuens T. Geroldi A. Origone P. Verdiani S. Cichero E. (2016). Molecular chaperones in the pathogenesis of amyotrophic lateral sclerosis: the role of HSPB1. *Hum. Mutat.* 37 1202–1208. 10.1002/humu.23062 27492805 PMC5108433

[B19] Carra S. (2009). The stress-inducible HspB8-Bag3 complex induces the eIF2alpha kinase pathway: implications for protein quality control and viral factory degradation? *Autophagy* 5 428–429. 10.4161/auto.5.3.7894 19202352

[B20] Carra S. Boncoraglio A. Kanon B. Brunsting J. F. Minoia M. Rana A. (2010). Identification of the *Drosophila* ortholog of HSPB8: implication of HSPB8 loss of function in protein folding diseases. *J. Biol. Chem.* 285 37811–37822. 10.1074/jbc.M110.127498 20858900 PMC2988385

[B21] Carra S. Rusmini P. Crippa V. Giorgetti E. Boncoraglio A. Cristofani R. (2013). Different anti-aggregation and pro-degradative functions of the members of the mammalian sHSP family in neurological disorders. *Philos. Trans. R. Soc. Lond. B Biol. Sci.* 368:20110409. 10.1098/rstb.2011.0409 23530259 PMC3638395

[B22] Carra S. Seguin S. J. Lambert H. Landry J. (2008a). HspB8 chaperone activity toward poly(Q)-containing proteins depends on its association with Bag3, a stimulator of macroautophagy. *J. Biol. Chem.* 283 1437–1444. 10.1074/jbc.M706304200 18006506

[B23] Carra S. Seguin S. J. Landry J. (2008b). HspB8 and Bag3: a new chaperone complex targeting misfolded proteins to macroautophagy. *Autophagy* 4 237–239. 10.4161/auto.5407 18094623

[B24] Carra S. Sivilotti M. Chavez Zobel A. T. Lambert H. Landry J. (2005). HspB8, a small heat shock protein mutated in human neuromuscular disorders, has in vivo chaperone activity in cultured cells. *Hum. Mol. Genet.* 14 1659–1669. 10.1093/hmg/ddi174 15879436

[B25] Castillo K. Nassif M. Valenzuela V. Rojas F. Matus S. Mercado G. (2013). Trehalose delays the progression of amyotrophic lateral sclerosis by enhancing autophagy in motoneurons. *Autophagy* 9 1308–1320. 10.4161/auto.25188 23851366

[B26] Chavez Zobel A. T. Loranger A. Marceau N. Theriault J. R. Lambert H. Landry J. (2003). Distinct chaperone mechanisms can delay the formation of aggresomes by the myopathy-causing R120G alphaB-crystallin mutant. *Hum. Mol. Genet.* 12 1609–1620. 10.1093/hmg/ddg173 12812987

[B27] Chou C. C. Zhang Y. Umoh M. E. Vaughan S. W. Lorenzini I. Liu F. (2018). TDP-43 pathology disrupts nuclear pore complexes and nucleocytoplasmic transport in ALS/FTD. *Nat. Neurosci.* 21 228–239. 10.1038/s41593-017-0047-3 29311743 PMC5800968

[B28] Chua J. P. Reddy S. L. Merry D. E. Adachi H. Katsuno M. Sobue G. (2014). Transcriptional activation of TFEB/ZKSCAN3 target genes underlies enhanced autophagy in spinobulbar muscular atrophy. *Hum. Mol. Genet.* 23 1376–1386. 10.1093/hmg/ddt527 24150846 PMC3919011

[B29] Cicardi M. E. Cristofani R. Rusmini P. Meroni M. Ferrari V. Vezzoli G. (2018). Tdp-25 routing to autophagy and proteasome ameliorates its aggregation in amyotrophic lateral sclerosis target cells. *Sci. Rep.* 8:12390. 10.1038/s41598-018-29658-2 30120266 PMC6098007

[B30] Ciechanover A. Kwon Y. T. (2015). Degradation of misfolded proteins in neurodegenerative diseases: therapeutic targets and strategies. *Exp. Mol. Med.* 47:e147. 10.1038/emm.2014.117 25766616 PMC4351408

[B31] Cook C. Petrucelli L. (2019). Genetic convergence brings clarity to the enigmatic red line in ALS. *Neuron* 101 1057–1069. 10.1016/j.neuron.2019.02.032 30897357

[B32] Cortes C. J. Ling S. C. Guo L. T. Hung G. Tsunemi T. Ly L. (2014a). Muscle expression of mutant androgen receptor accounts for systemic and motor neuron disease phenotypes in spinal and bulbar muscular atrophy. *Neuron* 82 295–307. 10.1016/j.neuron.2014.03.001 24742458 PMC4096235

[B33] Cortes C. J. Miranda H. C. Frankowski H. Batlevi Y. Young J. E. Le A. (2014b). Polyglutamine-expanded androgen receptor interferes with TFEB to elicit autophagy defects in SBMA. *Nat. Neurosci.* 17 1180–1189. 10.1038/nn.3787 25108912 PMC4180729

[B34] Crippa V. Boncoraglio A. Galbiati M. Aggarwal T. Rusmini P. Giorgetti E. (2013a). Differential autophagy power in the spinal cord and muscle of transgenic ALS mice. *Front Cell Neurosci* 7:234. 10.3389/fncel.2013.00234 24324403 PMC3840302

[B35] Crippa V. Galbiati M. Boncoraglio A. Rusmini P. Onesto E. Giorgetti E. (2013b). Motoneuronal and muscle-selective removal of ALS-related misfolded proteins. *Biochem. Soc. Trans.* 41 1598–1604. 10.1042/BST20130118 24256261

[B36] Crippa V. Cicardi M. E. Ramesh N. Seguin S. J. Ganassi M. Bigi I. (2016a). The chaperone HSPB8 reduces the accumulation of truncated TDP-43 species in cells and protects against TDP-43-mediated toxicity. *Hum. Mol. Genet.* 25 3908–3924. 10.1093/hmg/ddw232 27466192 PMC5291228

[B37] Crippa V. D’Agostino V. G. Cristofani R. Rusmini P. Cicardi M. E. Messi E. (2016b). Transcriptional induction of the heat shock protein B8 mediates the clearance of misfolded proteins responsible for motor neuron diseases. *Sci. Rep.* 6:22827. 10.1038/srep22827 26961006 PMC4785366

[B38] Crippa V. Sau D. Rusmini P. Boncoraglio A. Onesto E. Bolzoni E. (2010). The small heat shock protein B8 (HspB8) promotes autophagic removal of misfolded proteins involved in amyotrophic lateral sclerosis (ALS). *Hum. Mol. Genet.* 19 3440–3456. 10.1093/hmg/ddq257 20570967

[B39] Cristofani R. Crippa V. Rusmini P. Cicardi M. E. Meroni M. Licata N. V. (2017). Inhibition of retrograde transport modulates misfolded protein accumulation and clearance in motoneuron diseases. *Autophagy* 13 1280–1303. 10.1080/15548627.2017.1308985 28402699 PMC5584856

[B40] Cristofani R. Crippa V. Vezzoli G. Rusmini P. Galbiati M. Cicardi M. E. (2018). The small heat shock protein B8 (HSPB8) efficiently removes aggregating species of dipeptides produced in C9ORF72-related neurodegenerative diseases. *Cell Stress Chaperones* 23 1–12. 10.1007/s12192-017-0806-9 28608264 PMC5741577

[B41] Daoud H. Valdmanis P. N. Kabashi E. Dion P. Dupre N. Camu W. (2009). Contribution of TARDBP mutations to sporadic amyotrophic lateral sclerosis. *J. Med. Genet.* 46 112–114. 10.1136/jmg.2008.062463 18931000

[B42] Davies J. E. Sarkar S. Rubinsztein D. C. (2006). Trehalose reduces aggregate formation and delays pathology in a transgenic mouse model of oculopharyngeal muscular dystrophy. *Hum. Mol. Genet.* 15 23–31. 10.1093/hmg/ddi422 16311254

[B43] Du J. Liang Y. Xu F. Sun B. Wang Z. (2013). Trehalose rescues Alzheimer’s disease phenotypes in APP/PS1 transgenic mice. *J. Pharm. Pharmacol.* 65 1753–1756. 10.1111/jphp.12108 24236985

[B44] Elden A. C. Kim H. J. Hart M. P. Chen-Plotkin A. S. Johnson B. S. Fang X. (2010). Ataxin-2 intermediate-length polyglutamine expansions are associated with increased risk for ALS. *Nature* 466 1069–1075. 10.1038/nature09320 20740007 PMC2965417

[B45] El-Kadi A. M. Bros-Facer V. Deng W. Philpott A. Stoddart E. Banks G. (2010). The legs at odd angles (Loa) mutation in cytoplasmic dynein ameliorates mitochondrial function in SOD1G93A mouse model for motor neuron disease. *J. Biol. Chem.* 285 18627–18639. 10.1074/jbc.M110.129320 20382740 PMC2881788

[B46] Evans C. S. Holzbaur E. L. F. (2019). Autophagy and mitophagy in ALS. *Neurobiol. Dis.* 122 35–40. 10.1016/j.nbd.2018.07.005 29981842 PMC6366665

[B47] Fecto F. Yan J. Vemula S. P. Liu E. Yang Y. Chen W. (2011). SQSTM1 mutations in familial and sporadic amyotrophic lateral sclerosis. *Arch. Neurol.* 68 1440–1446. 10.1001/archneurol.2011.250 22084127

[B48] Fischbeck K. H. (1997). Kennedy disease. *J. Inherit. Metab. Dis.* 20 152–158. 9211187 10.1023/a:1005344403603

[B49] Fontaine J. M. Sun X. Hoppe A. D. Simon S. Vicart P. Welsh M. J. (2006). Abnormal small heat shock protein interactions involving neuropathy-associated HSP22 (HSPB8) mutants. *FASEB J.* 20 2168–2170. 10.1096/fj.06-5911fje 16935933

[B50] Freibaum B. D. Lu Y. Lopez-Gonzalez R. Kim N. C. Almeida S. Lee K. H. (2015). GGGGCC repeat expansion in C9orf72 compromises nucleocytoplasmic transport. *Nature* 525 129–133. 10.1038/nature14974 26308899 PMC4631399

[B51] Fuchs M. Luthold C. Guilbert S. M. Varlet A. A. Lambert H. Jette A. (2015). A role for the chaperone complex BAG3-HSPB8 in actin dynamics, spindle orientation and proper chromosome segregation during mitosis. *PLoS Genet.* 11:e1005582. 10.1371/journal.pgen.1005582 26496431 PMC4619738

[B52] Fuchs M. Poirier D. J. Seguin S. J. Lambert H. Carra S. Charette S. J. (2009). Identification of the key structural motifs involved in HspB8/HspB6-Bag3 interaction. *Biochem. J.* 425 245–255. 10.1042/BJ20090907 19845507

[B53] Fuchs M. Poirier D. J. Seguin S. J. Lambert H. Carra S. Charette S. J. (2010). Identification of the key structural motifs involved in HspB8/HspB6-Bag3 interaction. *Biochem. J.* 425 245–255. 10.1042/BJ20090907 19845507

[B54] Galbiati M. Crippa V. Rusmini P. Cristofani R. Cicardi M. E. Giorgetti E. (2014). ALS-related misfolded protein management in motor neurons and muscle cells. *Neurochem. Int.* 79 70–78. 10.1016/j.neuint.2014.10.007 25451799

[B55] Gamerdinger M. Carra S. Behl C. (2011a). Emerging roles of molecular chaperones and co-chaperones in selective autophagy: focus on BAG proteins. *J. Mol. Med.* 89 1175–1182. 10.1007/s00109-011-0795-6 21818581

[B56] Gamerdinger M. Kaya A. M. Wolfrum U. Clement A. M. Behl C. (2011b). BAG3 mediates chaperone-based aggresome-targeting and selective autophagy of misfolded proteins. *EMBO Rep.* 12 149–156. 10.1038/embor.2010.203 21252941 PMC3049430

[B57] Ganassi M. Mateju D. Bigi I. Mediani L. Poser I. Lee H. O. (2016). A surveillance function of the HSPB8-BAG3-HSP70 chaperone complex ensures stress granule integrity and dynamism. *Mol. Cell* 63 796–810. 10.1016/j.molcel.2016.07.021 27570075

[B58] Gasset-Rosa F. Lu S. Yu H. Chen C. Melamed Z. Guo L. (2019). Cytoplasmic TDP-43 de-mixing independent of stress granules drives inhibition of nuclear import, loss of nuclear TDP-43, and cell death. *Neuron* 102 339.e7–357.e7. 10.1016/j.neuron.2019.02.038 30853299 PMC6548321

[B59] Genis D. Ortega-Cubero S. San Nicolás H. Corral J. Gardenyes J. de Jorge L. (2018). Heterozygous *STUB1* mutation causes familial ataxia with cognitive affective syndrome (SCA48). *Neurology* 91 e1988–e1998. 10.1212/WNL.0000000000006550 30381368

[B60] Ghaoui R. Palmio J. Brewer J. Lek M. Needham M. Evila A. (2016). Mutations in HSPB8 causing a new phenotype of distal myopathy and motor neuropathy. *Neurology* 86 391–398. 10.1212/WNL.0000000000002324 26718575 PMC4776089

[B61] Giorgetti E. Rusmini P. Crippa V. Cristofani R. Boncoraglio A. Cicardi M. E. (2015). Synergic prodegradative activity of Bicalutamide and trehalose on the mutant androgen receptor responsible for spinal and bulbar muscular atrophy. *Hum. Mol. Genet.* 24 64–75. 10.1093/hmg/ddu419 25122660 PMC4262493

[B62] Gorter R. P. Stephenson J. Nutma E. Anink J. de Jonge J. C. Baron W. (2018). Rapidly progressive amyotrophic lateral sclerosis is associated with microglial reactivity and small heat shock protein expression in reactive astrocytes. *Neuropathol. Appl. Neurobiol.* 45 459–475. 10.1111/nan.12525 30346063 PMC7379307

[B63] Hageman J. Rujano M. A. van Waarde M. A. Kakkar V. Dirks R. P. Govorukhina N. (2010). A DNAJB chaperone subfamily with HDAC-dependent activities suppresses toxic protein aggregation. *Mol. Cell* 37 355–369. 10.1016/j.molcel.2010.01.001 20159555

[B64] Halievski K. Henley C. L. Domino L. Poort J. E. Fu M. Katsuno M. (2015). Androgen-dependent loss of muscle BDNF mRNA in two mouse models of SBMA. *Exp. Neurol.* 269 224–232. 10.1016/j.expneurol.2015.04.013 25929689 PMC4446172

[B65] Hartl F. U. Bracher A. Hayer-Hartl M. (2011). Molecular chaperones in protein folding and proteostasis. *Nature* 475 324–332. 10.1038/nature10317 21776078

[B66] Hayer S. N. Deconinck T. Bender B. Smets K. Zuchner S. Reich S. (2017). STUB1/CHIP mutations cause Gordon Holmes syndrome as part of a widespread multisystemic neurodegeneration: evidence from four novel mutations. *Orphanet J. Rare Dis.* 12:31. 10.1186/s13023-017-0580-x 28193273 PMC5307643

[B67] He Q. Koprich J. B. Wang Y. Yu W. B. Xiao B. G. Brotchie J. M. (2016). Treatment with trehalose prevents behavioral and neurochemical deficits produced in an AAV alpha-Synuclein rat model of Parkinson’s Disease. *Mol. Neurobiol.* 53 2258–2268. 10.1007/s12035-015-9173-7 25972237

[B68] Heilman P. L. Song S. Miranda C. J. Meyer K. Srivastava A. K. Knapp A. (2017). HSPB1 mutations causing hereditary neuropathy in humans disrupt non-cell autonomous protection of motor neurons. *Exp. Neurol.* 297 101–109. 10.1016/j.expneurol.2017.08.002 28797631 PMC5612892

[B69] Hishiya A. Salman M. N. Carra S. Kampinga H. H. Takayama S. (2011). BAG3 directly interacts with mutated alphaB-crystallin to suppress its aggregation and toxicity. *PLoS One* 6:e16828. 10.1371/journal.pone.0016828 21423662 PMC3057972

[B70] Irobi J. Almeida-Souza L. Asselbergh B. De Winter V. Goethals S. Dierick I. (2010). Mutant HSPB8 causes motor neuron-specific neurite degeneration. *Hum. Mol. Genet.* 19 3254–3265. 10.1093/hmg/ddq234 20538880 PMC2908473

[B71] Irobi J. Van Impe K. Seeman P. Jordanova A. Dierick I. Verpoorten N. (2004). Hot-spot residue in small heat-shock protein 22 causes distal motor neuropathy. *Nat. Genet.* 36 597–601. 10.1038/ng1328 15122253

[B72] Jaffer F. Murphy S. M. Scoto M. Healy E. Rossor A. M. Brandner S. (2012). BAG3 mutations: another cause of giant axonal neuropathy. *J. Peripher. Nerv. Syst.* 17 210–216. 10.1111/j.1529-8027.2012.00409.x 22734908

[B73] Jia B. Wu Y. Zhou Y. (2014). 14-3-3 and aggresome formation: implications in neurodegenerative diseases. *Prion* 8:28123. 24549097 10.4161/pri.28123 PMC4189886

[B74] Jovicic A. Mertens J. Boeynaems S. Bogaert E. Chai N. Yamada S. B. (2015). Modifiers of C9orf72 dipeptide repeat toxicity connect nucleocytoplasmic transport defects to FTD/ALS. *Nat. Neurosci.* 18 1226–1229. 10.1038/nn.4085 26308983 PMC4552077

[B75] Ju J. S. Fuentealba R. A. Miller S. E. Jackson E. Piwnica-Worms D. Baloh R. H. (2009). Valosin-containing protein (VCP) is required for autophagy and is disrupted in VCP disease. *J. Cell Biol.* 187 875–888. 10.1083/jcb.200908115 20008565 PMC2806317

[B76] Kakkar V. Meister-Broekema M. Minoia M. Carra S. Kampinga H. H. (2014). Barcoding heat shock proteins to human diseases: looking beyond the heat shock response. *Dis. Model. Mech.* 7 421–434. 10.1242/dmm.014563 24719117 PMC3974453

[B77] Kalmar B. Greensmith L. (2017). Cellular chaperones as therapeutic targets in ALS to restore protein homeostasis and improve cellular function. *Front. Mol. Neurosci.* 10:251. 10.3389/fnmol.2017.00251 28943839 PMC5596081

[B78] Kampinga H. H. Craig E. A. (2010). The HSP70 chaperone machinery: J proteins as drivers of functional specificity. *Nat. Rev. Mol. Cell Biol.* 11 579–592. 10.1038/nrm2941 20651708 PMC3003299

[B79] Kanack A. J. Newsom O. J. Scaglione K. M. (2018). Most mutations that cause spinocerebellar ataxia autosomal recessive type 16 (SCAR16) destabilize the protein quality-control E3 ligase CHIP. *J. Biol. Chem.* 293 2735–2743. 10.1074/jbc.RA117.000477 29317501 PMC5827432

[B80] Katsuno M. Adachi H. Doyu M. Minamiyama M. Sang C. Kobayashi Y. (2003). Leuprorelin rescues polyglutamine-dependent phenotypes in a transgenic mouse model of spinal and bulbar muscular atrophy. *Nat. Med.* 9 768–773. 10.1038/nm878 12754502

[B81] Katsuno M. Adachi H. Kume A. Li M. Nakagomi Y. Niwa H. (2002). Testosterone reduction prevents phenotypic expression in a transgenic mouse model of spinal and bulbar muscular atrophy. *Neuron* 35 843–854. 10.1016/s0896-6273(02)00834-6 12372280

[B82] Kieran D. Hafezparast M. Bohnert S. Dick J. R. Martin J. Schiavo G. (2005). A mutation in dynein rescues axonal transport defects and extends the life span of ALS mice. *J. Cell Biol.* 169 561–567. 10.1083/jcb.200501085 15911875 PMC2171702

[B83] Kim H. J. Taylor J. P. (2017). Lost in transportation: nucleocytoplasmic transport defects in ALS and other neurodegenerative diseases. *Neuron* 96 285–297. 10.1016/j.neuron.2017.07.029 29024655 PMC5678982

[B84] Klionsky D. J. Abdelmohsen K. Abe A. Abedin M. J. Abeliovich H. Acevedo Arozena A. (2016). Guidelines for the use and interpretation of assays for monitoring autophagy (3rd edition). *Autophagy* 12 1–222. 10.1080/15548627.2015.1100356 26799652 PMC4835977

[B85] Krishnan J. Lemmens R. Robberecht W. Van Den Bosch L. (2006). Role of heat shock response and Hsp27 in mutant SOD1-dependent cell death. *Exp. Neurol.* 200 301–310. 10.1016/j.expneurol.2006.02.135 16806187

[B86] Krishnan J. Vannuvel K. Andries M. Waelkens E. Robberecht W. Van Den Bosch L. (2008). Over-expression of Hsp27 does not influence disease in the mutant SOD1(G93A) mouse model of amyotrophic lateral sclerosis. *J. Neurochem.* 106 2170–2183. 10.1111/j.1471-4159.2008.05545.x 18624915

[B87] La Spada A. R. Wilson E. M. Lubahn D. B. Harding A. E. Fischbeck K. H. (1991). Androgen receptor gene mutations in X-linked spinal and bulbar muscular atrophy. *Nature* 352 77–79. 10.1038/352077a0 2062380

[B88] Lackie R. E. Maciejewski A. Ostapchenko V. G. Marques-Lopes J. Choy W. Y. Duennwald M. L. (2017). The Hsp70/Hsp90 chaperone machinery in neurodegenerative diseases. *Front. Neurosci.* 11:254. 10.3389/fnins.2017.00254 28559789 PMC5433227

[B89] Lee K. H. Zhang P. Kim H. J. Mitrea D. M. Sarkar M. Freibaum B. D. (2016). C9orf72 dipeptide repeats impair the assembly, dynamics, and function of membrane-less organelles. *Cell* 167 774.e17–788.e17. 10.1016/j.cell.2016.10.002 27768896 PMC5079111

[B90] Li Y. Guo Y. Wang X. Yu X. Duan W. Hong K. (2015). Trehalose decreases mutant SOD1 expression and alleviates motor deficiency in early but not end-stage amyotrophic lateral sclerosis in a SOD1-G93A mouse model. *Neuroscience* 298 12–25. 10.1016/j.neuroscience.2015.03.061 25841320

[B91] Lieberman A. P. Yu Z. Murray S. Peralta R. Low A. Guo S. (2014). Peripheral androgen receptor gene suppression rescues disease in mouse models of spinal and bulbar muscular atrophy. *Cell Rep.* 7 774–784. 10.1016/j.celrep.2014.02.008 24746732 PMC4356525

[B92] Lilienbaum A. (2013). Relationship between the proteasomal system and autophagy. *Int. J. Biochem. Mol. Biol.* 4 1–26. 23638318 PMC3627065

[B93] Lobsiger C. S. Boillee S. McAlonis-Downes M. Khan A. M. Feltri M. L. Yamanaka K. (2009). Schwann cells expressing dismutase active mutant SOD1 unexpectedly slow disease progression in ALS mice. *Proc. Natl. Acad. Sci. U.S.A.* 106 4465–4470. 10.1073/pnas.0813339106 19251638 PMC2657393

[B94] Mackenzie I. R. Nicholson A. M. Sarkar M. Messing J. Purice M. D. Pottier C. (2017). TIA1 mutations in amyotrophic lateral sclerosis and frontotemporal dementia promote phase separation and alter stress granule dynamics. *Neuron* 95 808.e9–816e9. 10.1016/j.neuron.2017.07.025 28817800 PMC5576574

[B95] Malena A. Pennuto M. Tezze C. Querin G. D’Ascenzo C. Silani V. (2013). Androgen-dependent impairment of myogenesis in spinal and bulbar muscular atrophy. *Acta Neuropathol.* 126 109–121. 10.1007/s00401-013-1122-9 23644820

[B96] Malik B. Devine H. Patani R. La Spada A. R. Hanna M. G. Greensmith L. (2019). Gene expression analysis reveals early dysregulation of disease pathways and links Chmp7 to pathogenesis of spinal and bulbar muscular atrophy. *Sci. Rep.* 9:3539. 10.1038/s41598-019-40118-3 30837566 PMC6401132

[B97] Mandrioli J. Crippa V. Cereda C. Bonetto V. Zucchi E. Gessani A. (2019). Proteostasis and ALS: protocol for a phase II, randomized, double blind, placebo controlled, multicenter clinical trial for Colchicine in ALS (Co-ALS). *BMJ Open* 9:e028486. 10.1136/bmjopen-2018-028486 31152038 PMC6549675

[B98] Marino M. Papa S. Crippa V. Nardo G. Peviani M. Cheroni C. (2015). Differences in protein quality control correlate with phenotype variability in 2 mouse models of familial amyotrophic lateral sclerosis. *Neurobiol. Aging* 36 492–504. 10.1016/j.neurobiolaging.2014.06.026 25085783

[B99] Mateju D. Franzmann T. M. Patel A. Kopach A. Boczek E. E. Maharana S. (2017). An aberrant phase transition of stress granules triggered by misfolded protein and prevented by chaperone function. *EMBO J.* 36 1669–1687. 10.15252/embj.201695957 28377462 PMC5470046

[B100] McCollum A. K. Casagrande G. Kohn E. C. (2009). Caught in the middle: the role of Bag3 in disease. *Biochem. J.* 425 e1–e3. 10.1042/BJ20091739 20001957 PMC7291699

[B101] Merabova N. Sariyer I. K. Saribas A. S. Knezevic T. Gordon J. Turco M. C. (2015). WW domain of BAG3 is required for the induction of autophagy in glioma cells. *J. Cell Physiol.* 230 831–841. 10.1002/jcp.24811 25204229 PMC4373658

[B102] Minoia M. Boncoraglio A. Vinet J. Morelli F. F. Brunsting J. F. Poletti A. (2014). BAG3 induces the sequestration of proteasomal clients into cytoplasmic puncta: implications for a proteasome-to-autophagy switch. *Autophagy* 10 1603–1621. 10.4161/auto.29409 25046115 PMC4206538

[B103] Molliex A. Temirov J. Lee J. Coughlin M. Kanagaraj A. P. Kim H. J. (2015). Phase separation by low complexity domains promotes stress granule assembly and drives pathological fibrillization. *Cell* 163 123–133. 10.1016/j.cell.2015.09.015 26406374 PMC5149108

[B104] Morelli F. F. Mediani L. Heldens L. Bertacchini J. Bigi I. Carra A. D. (2017). An interaction study in mammalian cells demonstrates weak binding of HSPB2 to BAG3, which is regulated by HSPB3 and abrogated by HSPB8. *Cell Stress Chaperones* 22 531–540. 10.1007/s12192-017-0769-x 28181153 PMC5465030

[B105] Musarò A. (2010). State of the art and the dark side of amyotrophic lateral sclerosis. *World J. Biol. Chem.* 26 62–68. 10.4331/wjbc.v1.i5.62 21540991 PMC3083964

[B106] Nagai M. Re D. B. Nagata T. Chalazonitis A. Jessell T. M. Wichterle H. (2007). Astrocytes expressing ALS-linked mutated SOD1 release factors selectively toxic to motor neurons. *Nat. Neurosci.* 10 615–622. 10.1038/nn1876 17435755 PMC3799799

[B107] Neumann M. Kwong L. K. Lee E. B. Kremmer E. Flatley A. Xu Y. (2009). Phosphorylation of S409/410 of TDP-43 is a consistent feature in all sporadic and familial forms of TDP-43 proteinopathies. *Acta Neuropathol.* 117 137–149. 10.1007/s00401-008-0477-9 19125255 PMC2693625

[B108] Neumann M. Sampathu D. M. Kwong L. K. Truax A. C. Micsenyi M. C. Chou T. T. (2006). Ubiquitinated TDP-43 in frontotemporal lobar degeneration and amyotrophic lateral sclerosis. *Science* 314 130–133. 10.1126/science.1134108 17023659

[B109] Nguyen D. K. H. Thombre R. Wang J. (2019). Autophagy as a common pathway in amyotrophic lateral sclerosis. *Neurosci. Lett.* 697 34–48. 10.1016/j.neulet.2018.04.006 29626651 PMC6170747

[B110] Nikoletopoulou V. Papandreou M. E. Tavernarakis N. (2015). Autophagy in the physiology and pathology of the central nervous system. *Cell Death Differ.* 22 398–407. 10.1038/cdd.2014.204 25526091 PMC4326580

[B111] Nivon M. Abou-Samra M. Richet E. Guyot B. Arrigo A. P. Kretz-Remy C. (2012). NF-kappaB regulates protein quality control after heat stress through modulation of the BAG3-HspB8 complex. *J. Cell Sci.* 125(Pt 5), 1141–1151. 10.1242/jcs.091041 22302993

[B112] Onesto E. Rusmini P. Crippa V. Ferri N. Zito A. Galbiati M. (2011). Muscle cells and motoneurons differentially remove mutant SOD1 causing familial amyotrophic lateral sclerosis. *J. Neurochem.* 118 266–280. 10.1111/j.1471-4159.2011.07298.x 21554318 PMC3206220

[B113] Oskarsson B. Gendron T. F. Staff N. P. (2018). Amyotrophic lateral sclerosis: an update for 2018. *Mayo Clin. Proc.* 93 1617–1628. 10.1016/j.mayocp.2018.04.007 30401437

[B114] Pakdaman Y. Sanchez-Guixe M. Kleppe R. Erdal S. Bustad H. J. Bjorkhaug L. (2017). In vitro characterization of six STUB1 variants in spinocerebellar ataxia 16 reveals altered structural properties for the encoded CHIP proteins. *Biosci. Rep.* 37:BSR20170251. 10.1042/BSR20170251 28396517 PMC5408658

[B115] Patel A. Lee H. O. Jawerth L. Maharana S. Jahnel M. Hein M. Y. (2015). A liquid-to-solid phase transition of the ALS protein FUS accelerated by disease mutation. *Cell* 162 1066–1077. 10.1016/j.cell.2015.07.047 26317470

[B116] Patel Y. J. Payne Smith M. D. de Belleroche J. Latchman D. S. (2005). Hsp27 and Hsp70 administered in combination have a potent protective effect against FALS-associated SOD1-mutant-induced cell death in mammalian neuronal cells. *Brain Res. Mol. Brain Res.* 134 256–274. 10.1016/j.molbrainres.2004.10.028 15836922

[B117] Perera N. D. Sheean R. K. Lau C. L. Shin Y. S. Beart P. M. Horne M. K. (2018). Rilmenidine promotes MTOR-independent autophagy in the mutant SOD1 mouse model of amyotrophic lateral sclerosis without slowing disease progression. *Autophagy* 14 534–551. 10.1080/15548627.2017.1385674 28980850 PMC5915012

[B118] Perucho J. Casarejos M. J. Gomez A. Solano R. M. de Yebenes J. G. Mena M. A. (2012). Trehalose protects from aggravation of amyloid pathology induced by isoflurane anesthesia in APP(swe) mutant mice. *Curr. Alzheimer Res.* 9 334–343. 10.2174/156720512800107573 22272607

[B119] Philips T. Bento-Abreu A. Nonneman A. Haeck W. Staats K. Geelen V. (2013). Oligodendrocyte dysfunction in the pathogenesis of amyotrophic lateral sclerosis. *Brain* 136(Pt 2), 471–482. 10.1093/brain/aws339 23378219 PMC3572934

[B120] Philips T. Robberecht W. (2011). Neuroinflammation in amyotrophic lateral sclerosis: role of glial activation in motor neuron disease. *Lancet Neurol.* 10 253–263. 10.1016/S1474-4422(11)70015-1 21349440

[B121] Piccolella M. Crippa V. Cristofani R. Rusmini P. Galbiati M. Cicardi M. E. (2017). The small heat shock protein B8 (HSPB8) modulates proliferation and migration of breast cancer cells. *Oncotarget* 8 10400–10415. 10.18632/oncotarget.14422 28060751 PMC5354667

[B122] Poletti A. (2004). The polyglutamine tract of androgen receptor: from functions to dysfunctions in motor neurons. *Front. Neuroendocrinol.* 25:1–26. 10.1016/j.yfrne.2004.03.001 15183036

[B123] Rauch J. N. Tse E. Freilich R. Mok S. A. Makley L. N. Southworth D. R. (2017). BAG3 is a modular, scaffolding protein that physically links heat shock protein 70 (Hsp70) to the small heat shock proteins. *J. Mol. Biol.* 429 128–141. 10.1016/j.jmb.2016.11.013 27884606 PMC5186407

[B124] Robberecht W. Philips T. (2013). The changing scene of amyotrophic lateral sclerosis. *Nat. Rev. Neurosci.* 14 248–264. 10.1038/nrn3430 23463272

[B125] Rocchi A. Milioto C. Parodi S. Armirotti A. Borgia D. Pellegrini M. (2016). Glycolytic-to-oxidative fiber-type switch and mTOR signaling activation are early-onset features of SBMA muscle modified by high-fat diet. *Acta Neuropathol.* 132 127–144. 10.1007/s00401-016-1550-4 26971100 PMC4911374

[B126] Rodriguez-Navarro J. A. Rodriguez L. Casarejos M. J. Solano R. M. Gomez A. Perucho J. (2010). Trehalose ameliorates dopaminergic and tau pathology in parkin deleted/tau overexpressing mice through autophagy activation. *Neurobiol. Dis.* 39 423–438. 10.1016/j.nbd.2010.05.014 20546895

[B127] Rusmini P. Bolzoni E. Crippa V. Onesto E. Sau D. Galbiati M. (2010). Proteasomal and autophagic degradative activities in spinal and bulbar muscular atrophy. *Neurobiol. Dis.* 40 361–369. 10.1016/j.nbd.2010.06.016 20621188

[B128] Rusmini P. Cortese K. Crippa V. Cristofani R. Cicardi M. E. Ferrari V. (2019). Trehalose induces autophagy via lysosomal-mediated TFEB activation in models of motoneuron degeneration. *Autophagy* 15 631–651. 10.1080/15548627.2018.1535292 30335591 PMC6526812

[B129] Rusmini P. Crippa V. Giorgetti E. Boncoraglio A. Cristofani R. Carra S. (2013). Clearance of the mutant androgen receptor in motoneuronal models of spinal and bulbar muscular atrophy. *Neurobiol. Aging* 34 2585–2603. 10.1016/j.neurobiolaging.2013.05.026 23810450 PMC3748343

[B130] Rusmini P. Cristofani R. Galbiati M. Cicardi M. E. Meroni M. Ferrari V. (2017). The role of the heat shock protein B8 (HSPB8) in motoneuron diseases. *Front. Mol. Neurosci.* 10:176. 10.3389/fnmol.2017.00176 28680390 PMC5478700

[B131] Rusmini P. Polanco M. J. Cristofani R. Cicardi M. E. Meroni M. Galbiati M. (2015). Aberrant autophagic response in the muscle of a knock-in mouse model of spinal and bulbar muscular atrophy. *Sci. Rep.* 5:15174. 10.1038/srep15174 26490709 PMC4614888

[B132] Sahashi K. Katsuno M. Hung G. Adachi H. Kondo N. Nakatsuji H. (2015). Silencing neuronal mutant androgen receptor in a mouse model of spinal and bulbar muscular atrophy. *Hum. Mol. Genet.* 24 5985–5994. 10.1093/hmg/ddv300 26231218

[B133] Sanbe A. Daicho T. Mizutani R. Endo T. Miyauchi N. Yamauchi J. (2009). Protective effect of geranylgeranylacetone via enhancement of HSPB8 induction in desmin-related cardiomyopathy. *PLoS One* 4:e5351. 10.1371/journal.pone.0005351 19399179 PMC2670514

[B134] Sandell S. Huovinen S. Palmio J. Raheem O. Lindfors M. Zhao F. (2016). Diagnostically important muscle pathology in DNAJB6 mutated LGMD1D. *Acta Neuropathol. Commun.* 4:9. 10.1186/s40478-016-0276-9 26847086 PMC4743201

[B135] Sarkar S. Chigurupati S. Raymick J. Mann D. Bowyer J. F. Schmitt T. (2014). Neuroprotective effect of the chemical chaperone, trehalose in a chronic MPTP-induced Parkinson’s disease mouse model. *Neurotoxicology* 44 250–262. 10.1016/j.neuro.2014.07.006 25064079

[B136] Sarparanta J. Jonson P. H. Golzio C. Sandell S. Luque H. Screen M. (2012). Mutations affecting the cytoplasmic functions of the co-chaperone DNAJB6 cause limb-girdle muscular dystrophy. *Nat. Genet.* 44 S451–S452. 10.1038/ng.1103 22366786 PMC3315599

[B137] Sato T. Hayashi Y. K. Oya Y. Kondo T. Sugie K. Kaneda D. (2013). DNAJB6 myopathy in an Asian cohort and cytoplasmic/nuclear inclusions. *Neuromuscul. Disord.* 23 269–276. 10.1016/j.nmd.2012.12.010 23394708

[B138] Schaeffer V. Goedert M. (2012). Stimulation of autophagy is neuroprotective in a mouse model of human tauopathy. *Autophagy* 8 1686–1687. 10.4161/auto.21488 22874558 PMC3494601

[B139] Seguin S. J. Morelli F. F. Vinet J. Amore D. De Biasi S. Poletti A. (2014). Inhibition of autophagy, lysosome and VCP function impairs stress granule assembly. *Cell Death Differ.* 21 1838–1851. 10.1038/cdd.2014.103 25034784 PMC4227144

[B140] Seidel K. Vinet J. Dunnen W. F. Brunt E. R. Meister M. Boncoraglio A. (2012). The HSPB8-BAG3 chaperone complex is upregulated in astrocytes in the human brain affected by protein aggregation diseases. *Neuropathol. Appl. Neurobiol.* 38 39–53. 10.1111/j.1365-2990.2011.01198.x 21696420

[B141] Selcen D. Muntoni F. Burton B. K. Pegoraro E. Sewry C. Bite A. V. (2009). Mutation in BAG3 causes severe dominant childhood muscular dystrophy. *Ann. Neurol.* 65 83–89. 10.1002/ana.21553 19085932 PMC2639628

[B142] Senft D. Ronai Z. A. (2015). UPR, autophagy, and mitochondria crosstalk underlies the ER stress response. *Trends Biochem. Sci.* 40 141–148. 10.1016/j.tibs.2015.01.002 25656104 PMC4340752

[B143] Sharp P. S. Akbar M. T. Bouri S. Senda A. Joshi K. Chen H. J. (2008). Protective effects of heat shock protein 27 in a model of ALS occur in the early stages of disease progression. *Neurobiol. Dis.* 30 42–55. 10.1016/j.nbd.2007.12.002 18255302

[B144] Shi C. H. Rubel C. Soss S. E. Sanchez-Hodge R. Zhang S. Madrigal S. C. (2018). Disrupted structure and aberrant function of CHIP mediates the loss of motor and cognitive function in preclinical models of SCAR16. *PLoS Genet.* 14:e1007664. 10.1371/journal.pgen.1007664 30222779 PMC6160236

[B145] Shi Y. Wang J. Li J. D. Ren H. Guan W. He M. (2013). Identification of CHIP as a novel causative gene for autosomal recessive cerebellar ataxia. *PLoS One* 8:e81884. 10.1371/journal.pone.0081884 24312598 PMC3846781

[B146] Simeoni S. Mancini M. A. Stenoien D. L. Marcelli M. Weigel N. L. Zanisi M. (2000). Motoneuronal cell death is not correlated with aggregate formation of androgen receptors containing an elongated polyglutamine tract. *Hum. Mol. Genet.* 9 133–144. 10.1093/hmg/9.1.133 10587588

[B147] Soraru G. D’Ascenzo C. Polo A. Palmieri A. Baggio L. Vergani L. (2008). Spinal and bulbar muscular atrophy: skeletal muscle pathology in male patients and heterozygous females. *J. Neurol. Sci.* 264 100–105. 10.1016/j.jns.2007.08.012 17854832

[B148] Stenoien D. L. Cummings C. J. Adams H. P. Mancini M. G. Patel K. DeMartino G. N. (1999). Polyglutamine-expanded androgen receptors form aggregates that sequester heat shock proteins, proteasome components and SRC-1, and are suppressed by the HDJ-2 chaperone. *Hum. Mol. Genet.* 8 731–741. 10.1093/hmg/8.5.731 10196362

[B149] Sun X. Fontaine J. M. Bartl I. Behnam B. Welsh M. J. Benndorf R. (2007). Induction of Hsp22 (HspB8) by estrogen and the metalloestrogen cadmium in estrogen receptor-positive breast cancer cells. *Cell Stress Chaperones* 12 307–319. 10.1379/CSC-276.1 18229450 PMC2134793

[B150] Takayama S. Reed J. C. (2001). Molecular chaperone targeting and regulation by BAG family proteins. *Nat. Cell Biol.* 3 E237–E241. 10.1038/ncb1001-e237 11584289

[B151] Tanaka M. Machida Y. Niu S. Ikeda T. Jana N. R. Doi H. (2004). Trehalose alleviates polyglutamine-mediated pathology in a mouse model of Huntington disease. *Nat. Med.* 10 148–154. 10.1038/nm985 14730359

[B152] Taylor J. P. Brown R. H. Jr. Cleveland D. W. (2016). Decoding ALS: from genes to mechanism. *Nature* 539 197–206. 10.1038/nature20413 27830784 PMC5585017

[B153] Teyssou E. Takeda T. Lebon V. Boillee S. Doukoure B. Bataillon G. (2013). Mutations in SQSTM1 encoding p62 in amyotrophic lateral sclerosis: genetics and neuropathology. *Acta Neuropathol.* 125 511–522. 10.1007/s00401-013-1090-0 23417734

[B154] Tresse E. Salomons F. A. Vesa J. Bott L. C. Kimonis V. Yao T. P. (2010). VCP/p97 is essential for maturation of ubiquitin-containing autophagosomes and this function is impaired by mutations that cause IBMPFD. *Autophagy* 6 217–227. 10.4161/auto.6.2.11014 20104022 PMC2929010

[B155] Trotti D. Rolfs A. Danbolt N. C. Brown R. H. Jr. Hediger M. A. (1999). SOD1 mutants linked to amyotrophic lateral sclerosis selectively inactivate a glial glutamate transporter. *Nat. Neurosci.* 2 427–433. 10.1038/12227 10321246

[B156] Turner B. J. Ackerley S. Davies K. E. Talbot K. (2010). Dismutase-competent SOD1 mutant accumulation in myelinating Schwann cells is not detrimental to normal or transgenic ALS model mice. *Hum. Mol. Genet.* 19 815–824. 10.1093/hmg/ddp550 20008901

[B157] Ulbricht A. Arndt V. Hohfeld J. (2013). Chaperone-assisted proteostasis is essential for mechanotransduction in mammalian cells. *Commun. Integr. Biol.* 6:e24925. 10.4161/cib.24925 23986815 PMC3737759

[B158] Ulbricht A. Gehlert S. Leciejewski B. Schiffer T. Bloch W. Hohfeld J. (2015). Induction and adaptation of chaperone-assisted selective autophagy CASA in response to resistance exercise in human skeletal muscle. *Autophagy* 11 538–546. 10.1080/15548627.2015.1017186 25714469 PMC4502687

[B159] Valenzuela V. Nassif M. Hetz C. (2018). Unraveling the role of motoneuron autophagy in ALS. *Autophagy* 14 733–737. 10.1080/15548627.2018.1432327 29388464 PMC5959335

[B160] Verma R. Oania R. S. Kolawa N. J. Deshaies R. J. (2013). Cdc48/p97 promotes degradation of aberrant nascent polypeptides bound to the ribosome. *eLife* 2:e00308. 10.7554/eLife.00308 23358411 PMC3552423

[B161] Villa A. Vegeto E. Poletti A. Maggi A. (2016). Estrogens, neuroinflammation, and neurodegeneration. *Endocr. Rev.* 37 372–402. 10.1210/er.2016-1007 27196727 PMC4971309

[B162] Vleminckx V. Van Damme P. Goffin K. Delye H. Van Den Bosch L. Robberecht W. (2002). Upregulation of HSP27 in a transgenic model of ALS. *J. Neuropathol. Exp. Neurol.* 61 968–974. 10.1093/jnen/61.11.968 12430713

[B163] Wang J. Slunt H. Gonzales V. Fromholt D. Coonfield M. Copeland N. G. (2003). Copper-binding-site-null SOD1 causes ALS in transgenic mice: aggregates of non-native SOD1 delineate a common feature. *Hum. Mol. Genet.* 12 2753–2764. 10.1093/hmg/ddg312 12966034

[B164] Wilhelmus M. M. Boelens W. C. Otte-Holler I. Kamps B. Kusters B. Maat-Schieman M. L. (2006). Small heat shock protein HspB8: its distribution in Alzheimer’s disease brains and its inhibition of amyloid-beta protein aggregation and cerebrovascular amyloid-beta toxicity. *Acta Neuropathol.* 111 139–149. 10.1007/s00401-005-0030-z 16485107

[B165] Woerner A. C. Frottin F. Hornburg D. Feng L. R. Meissner F. Patra M. (2016). Cytoplasmic protein aggregates interfere with nucleocytoplasmic transport of protein and RNA. *Science* 351 173–176. 10.1126/science.aad2033 26634439

[B166] Xilouri M. Stefanis L. (2015). Chaperone mediated autophagy to the rescue: a new-fangled target for the treatment of neurodegenerative diseases. *Mol. Cell Neurosci.* 66(Pt A), 29–36. 10.1016/j.mcn.2015.01.003 25724482

[B167] Xu Y. Halievski K. Henley C. Atchison W. D. Katsuno M. Adachi H. (2016). Defects in neuromuscular transmission may underlie motor dysfunction in spinal and bulbar muscular atrophy. *J. Neurosci.* 36 5094–5106. 10.1523/JNEUROSCI.3485-15.2016 27147661 PMC4854970

[B168] Xu Y. Halievski K. Katsuno M. Adachi H. Sobue G. Breedlove S. M. (2018). Pre-clinical symptoms of SBMA may not be androgen-dependent: implications from two SBMA mouse models. *Hum. Mol. Genet.* 27 2425–2442. 10.1093/hmg/ddy142 29897452 PMC6030880

[B169] Xu Z. Graham K. Foote M. Liang F. Rizkallah R. Hurt M. (2013). 14-3-3 protein targets misfolded chaperone-associated proteins to aggresomes. *J. Cell Sci.* 126(Pt 18), 4173–4186. 10.1242/jcs.126102 23843611 PMC3772389

[B170] Yerbury J. J. Gower D. Vanags L. Roberts K. Lee J. A. Ecroyd H. (2013). The small heat shock proteins alphaB-crystallin and Hsp27 suppress SOD1 aggregation in vitro. *Cell Stress Chaperones* 18 251–257. 10.1007/s12192-012-0371-1 22993064 PMC3581626

[B171] Yew E. H. Cheung N. S. Choy M. S. Qi R. Z. Lee A. Y. Peng Z. F. (2005). Proteasome inhibition by lactacystin in primary neuronal cells induces both potentially neuroprotective and pro-apoptotic transcriptional responses: a microarray analysis. *J. Neurochem.* 94 943–956. 10.1111/j.1471-4159.2005.03220.x 15992382

[B172] Zhang F. Strom A. L. Fukada K. Lee S. Hayward L. J. Zhu H. (2007). Interaction between familial amyotrophic lateral sclerosis (ALS)-linked SOD1 mutants and the dynein complex. *J. Biol. Chem.* 282 16691–16699. 10.1074/jbc.M609743200 17403682

[B173] Zhang P. Fan B. Yang P. Temirov J. Messing J. Kim H. J. (2019). Chronic optogenetic induction of stress granules is cytotoxic and reveals the evolution of ALS-FTD pathology. *eLife* 8:e39578. 10.7554/eLife.39578 30893049 PMC6426440

[B174] Zhang X. Chen S. Song L. Tang Y. Shen Y. Jia L. (2014). MTOR-independent, autophagic enhancer trehalose prolongs motor neuron survival and ameliorates the autophagic flux defect in a mouse model of amyotrophic lateral sclerosis. *Autophagy* 10 588–602. 10.4161/auto.27710 24441414 PMC4091147

[B175] Zhang X. Li L. Chen S. Yang D. Wang Y. Zhang X. (2011). Rapamycin treatment augments motor neuron degeneration in SOD1(G93A) mouse model of amyotrophic lateral sclerosis. *Autophagy* 7 412–425. 21193837 10.4161/auto.7.4.14541

